# Loneliness and Well-Being During the Covid-19 Pandemic: Associations with Personality and Emotion Regulation

**DOI:** 10.1007/s10902-020-00326-5

**Published:** 2020-10-20

**Authors:** Danièle A. Gubler, Lisa M. Makowski, Stefan J. Troche, Katja Schlegel

**Affiliations:** Institute for Psychology, University of Bern, Fabrikstr. 8, 3012 Bern, Switzerland

**Keywords:** Loneliness, Well-being, Neuroticism, Extraversion, Emotion regulation

## Abstract

The present study examined how neuroticism, extraversion, and emotion regulation were related to loneliness and well-being during 6 weeks of major public life restrictions in the Covid-19 pandemic in Switzerland. Cross-sectional results from 466 participants showed that neuroticism and emotion regulation strategies were associated with higher loneliness and lower well-being. However, in contrast to prior research, associations of extraversion with loneliness and well-being were weak and were qualified by interactions with emotion regulation. For introverts, maladaptive cognitive strategies such as rumination or catastrophizing were related to higher levels of loneliness. For extraverts, emotion suppression was related to lower levels of affective well-being. Individuals with low maladaptive regulation reported higher well-being the longer the public life restrictions were in place at the time of study participation. These findings suggest that first, extraversion may lose some of its protective value for loneliness and well-being when opportunities to engage in social activities are limited; second, that loneliness and well-being do not decrease over 6 weeks of public life restrictions; and third, that future studies should further investigate the moderating role of emotion regulation on the link between personality, loneliness, and well-being.

## Introduction

On March 11, 2020, the World Health Organization (WHO) classified the Covid-19 (coronavirus disease) outbreak as a pandemic (WHO 2020a). In an attempt to slow down the spread of the virus, serious public life restriction measures were taken in many countries around the world, including the temporary closure of businesses, shops, restaurants, schools, as well as entertainment and leisure facilities. Many governments urged their citizens to stay at home or implemented lockdown measures allowing only those with important reasons (buying groceries or medication, going to work) to leave the house. As noted by researchers (e.g., Folk et al. 2020; Holmes et al. 2020), the media, and international organizations (e.g., WHO 2020b), these disruptive measures and the pandemic in general have resulted in severe psychological stress for many individuals around the world due to worries over health, financial security, or adaptation to new living conditions. Social distancing also may pose a great challenge to many, as friends and relatives can no longer be visited and physical contact should be avoided (Brooks et al. 2020). In summary, most people were dealing with an unprecedented burden of unknown duration (Altena et al. 2020; Holmes et al. 2020).

Previous research has shown that isolation (even for health reasons as in the case of quarantine) can lead to various psychological problems such as enhanced feelings of loneliness and reduced well-being (Brooks et al. 2020). Loneliness is defined as the subjective feeling of disconnectedness representing the discrepancy between desired and actual social relationships (Buecker et al. 2020; Hawkley and Cacioppo 2010). Thus, loneliness refers to perceived social isolation, which does not depend on objective criteria but on subjective feelings (Hawkley and Cacioppo 2010). Well-being, in turn, is defined as a multifaceted construct containing cognitive (evaluative judgement of life satisfaction) and affective aspects (i.e., positive and negative emotions; Diener et al. 2003). Well-being has further been compared to a seesaw oscillating between personal resources and life challenges (Dodge et al. 2012). Accordingly, stable well-being is achieved when the individual has the psychological, social and physical resources needed to meet a particular psychological, social and/or physical challenge (Dodge et al. 2012).

Differences in long-term levels of loneliness and well-being are strongly associated with and predicted by stable personality traits (Buecker et al. 2020; Modersitzki et al. 2020; Ozer and Benet-Martinez 2006). From the perspective of the five-factor model of personality (FFM; Costa and McCrae 1980), the most consistent relationships with loneliness and well-being were found for neuroticism and extraversion (Buecker et al. 2020; DeNeve and Cooper 1998; Diener et al. 2003; Modersitzki et al. 2020; Steel et al. 2008). Neuroticism is characterized by a vulnerability to negative feelings and greater distress in changing life situations (Buecker et al. 2020; Kroencke et al. 2020; Liu et al. 2021; Suls and Martin 2005). Emotionally unstable individuals (i.e., individuals high on neuroticism) experience fear, depression, and guilt more often than emotionally stable individuals and are more sensitive to cues of social rejection (Denissen and Penke 2008). Emotionally unstable individuals also have more dysfunctional interpersonal relationships and are less satisfied with their relationships (Vater and Schröder-Abé 2015). Among the five factors of personality, neuroticism has been demonstrated to be the strongest correlate for reduced well-being and enhanced loneliness (Albuquerque et al. 2012; Buecker et al. 2020), which has also been confirmed in recent Covid-19-related studies (Aschwanden et al. 2020; Modersitzki et al. 2020).

Extraversion, in turn, is associated with engaging in and enjoying social interactions (John et al. 2008; Srivastava et al. 2008), participating in social activities (Lee et al. 2008), and greater social networks (Harris et al. 2017; Roberts et al. 2008). Extraverts receive greater social support from others (Asendorpf and Wilpers 1998), suggesting that they can rely on more social support in times of crisis. Moreover, extraverts show more positive affect and maintain their positive affect longer than introverts, especially when confronted with emotionally ambivalent situations (Steel et al. 2008). Extraverts therefore generally experience less loneliness and more well-being compared to introverts, with medium to high effect sizes (Albuquerque et al. 2012; Buecker et al. 2020).

Subjective feelings of loneliness and well-being are not only associated with basic personality traits, but also with how well a person is able to deal with emotional situations and regulate his or her own feelings (Kearns and Creaven 2017; Nyklíček et al. 2010). Researchers have identified various cognitive and behavioral strategies for emotion regulation that can broadly be divided into adaptive and maladaptive strategies (Garnefski and Kraaij 2006; Gross 2013; Gross and John 2003; Thompson 1991). Strategies like acceptance, cognitive reappraisal, refocusing on positive aspects of the situation, or focusing on finding a solution for a problem are typically considered adaptive in that they help downregulating negative emotions quickly (Garnefski and Kraaij 2006; Gross and John 2003). Suppressing emotions (i.e., not expressing them to others), self-blame, rumination, and catastrophizing are considered maladaptive regulation strategies because they may prolong or even deepen felt negative emotions (Garnefski and Kraaij 2006; Gross and John 2003). While suppression is considered a behavioral strategy that modifies one’s response to a felt (negative) emotion, the other strategies operate at an earlier, cognitive level and can change the felt emotion itself (Gross and John 2003). Research has demonstrated that the habitual use of adaptive strategies is related to greater well-being and reduced loneliness (Kearns and Creaven 2017; Quoidbach et al. 2010). Using maladaptive strategies, on the contrary, is associated with lower well-being and depression (Verzeletti et al. 2016).

Personality traits and emotion regulation strategies are interrelated. Neuroticism goes along with the use of maladaptive strategies such as rumination, worry, and avoidance, whereas extraversion is associated with using more adaptive strategies such as reappraisal, problem solving, or acceptance (see meta-analysis by Barańczuk 2019). Generally, the association with emotion regulation is stronger for neuroticism, with effect sizes being moderate to large, than for extraversion, where effect sizes are small to moderate (Barańczuk 2019). It is thus conceivable that personality traits and emotion regulation styles interact with each other in predicting outcomes such as well-being. For example, Cabello and Fernandez-Berrocal (2015) demonstrated that adaptive emotion regulation strategies reduced the negative effects of introversion and enhanced the positive effects of extraversion on well-being. Moreover, Hasking and colleagues (2010) found that neuroticism was more strongly associated with self-injury when participants also reported high levels of emotional suppression. However, studies examining interactions between neuroticism, extraversion, and emotion regulation remain rare.

### The Present Study

Most studies on personality traits and emotion regulation strategies as correlates of loneliness and well-being focused on long-lasting and stable associations without considering specific life contexts and temporal change (Diener et al. 2003). The Covid-19 pandemic therefore provides a unique context to test how personality and emotion regulation are associated with the subjective feeling of loneliness and well-being during a time in which “normal” life is disrupted for most people due to imposed social and public life restrictions. Moreover, with restrictions lasting for weeks or even months, it is also possible to test how the association of personality and emotion regulation with well-being and loneliness changes over time.

In the present study, we asked people living in Switzerland to evaluate their stable personality traits and emotional regulation strategies, as well as their loneliness and affective well-being during the last 7 days. To test for temporal changes in the associations between these variables, cross-sectional data was collected over a period of 6 weeks in March and April 2020 during which most shops, businesses, and restaurants were closed and gatherings of more than five people were banned (see methods for details). Swiss citizens were urged to work from home and stay at home as much as possible. Although people were allowed to leave their home, physical social interactions were massively restricted.

Based on the literature presented above, we had the following assumptions. First, with respect to personality, we hypothesized neuroticism to be negatively associated with well-being and positively associated with loneliness, as emotionally unstable individual are characterized by an increased vulnerability to stress and changes in life situations (Albuquerque et al. 2012; Buecker et al. 2020). Our expectations regarding extraversion were less clear. On the one hand, given that many studies found positive links between extraversion and well-being, this association may also be expected to hold during the Covid-19 pandemic. For instance, extraverts may feel less lonely and stressed because they have access to more social support (Asendorpf and Wilpers 1998). On the other hand, it is also conceivable that extraverts are more negatively affected by social distancing policies than introverts. Since introverts are less likely to engage in social interactions than extraverts (John et al. 2008; Srivastava et al. 2008), social distancing requirements may have a smaller impact on their lives (Folk et al. 2020; Kette 1991).

Second, regarding emotion regulation strategies, we hypothesized that individuals reporting a high habitual use of maladaptive strategies such as suppression or rumination would experience more loneliness and reduced well-being in line with past studies (e.g., Verzeletti et al. 2016). Specifically, individuals who suppress (i.e., do not express to others) their anxiety or worries during the generally challenging lockdown period may be less likely to communicate or share their experiences with others and may therefore feel more burdened and lonely. In addition, the pandemic and its detrimental economic and social consequences may provide a lot of potential to ruminate and catastrophize for individuals that are prone to using maladaptive strategies, likely leading to lower well-being. In contrast, the use of adaptive strategies such as reappraisal, acceptance, or focusing on planning may help re-organizing one’s everyday life during the lockdown in a way that is beneficial for one’s well-being.

We also investigated whether the interplay of personality and emotion regulation affected loneliness and well-being beyond simple main effects. Because literature on potential interaction effects is scarce, we looked at these relationships in an explorative manner. Thereby, we defined two key aspects of interest: First, we examined whether emotion regulation styles would more likely interact with extraversion than neuroticism (see Cabello and Fernandez-Berrocal 2015), since extraversion shows lower direct associations with emotion regulation (Barańczuk 2019). If the everyday social life of extraverts is indeed more strongly affected during the pandemic than that of introverts, it might for instance be that adaptive emotion regulation matters more strongly in extraverts for buffering the effects of lockdown measures. Second, we examined whether emotion regulation strategies interacted with each other in their relationship with psychological well-being. This is warranted because adaptive regulation strategies, maladaptive strategies, and emotional suppression are not substantially correlated with each other and there is some evidence that certain combinations (e.g., high values on both maladaptive and adaptive strategies) are particularly beneficial or detrimental to well-being (Chesney and Gordon 2017; Dixon-Gordon et al. 2015).

Our last expectation was about the influence of time spent under public life restrictions on loneliness and well-being. Since some studies demonstrated that emotional distress increases with the length of quarantine (Hawryluck et al. 2004), we expected people who participated in the present study at a later stage (within the 6 weeks of data collection) to experience more loneliness and less well-being. Again, we also explored possible interactions with personality and emotion regulation. It might for example be that well-being is only impaired when a person lacks adaptive regulation strategies.

The present study was not preregistered.

## Method

### Sample and Procedure

The sample consisted of *N* = 466 participants (79.6% female) residing in Switzerland with a mean age of 31.7 (± 16.2). Fifteen participants were excluded because they did not reside in Switzerland and may not have been subject to the same Covid-19 restrictions. Data collection took place from March 26 until April 27, 2020, and each participant completed the survey only once during this 6-week period. By the start of data collection, people in Switzerland had lived under the public life restrictions for about 10 days. Specifically, Switzerland declared a state of emergency on March 16, closed non-essential shops, businesses, schools, and restaurants, and prohibited gatherings of more than five individuals. People were urged to stay at home as much as possible and only to go outside for important reasons such as buying groceries or visiting a doctor. These measures remained active for the whole duration of data collection. On the 27th of April, the first loosening of the restrictions took place.

Participants were recruited via the participant pool of the University of Bern, online advertisements through Facebook, and the University of Zurich for Senior Citizens. Psychology students from the participant pool (*N* = 210) received one credit point for their participation, the other participants (*N* = 256) took part without compensation. The study was approved by the local ethics committee and was administrated online through Qualtrics (Qualtrics, Provo, UT). Participants gave their informed consent before completing the questionnaires described below. Further demographic information is provided in Table 1.

**Table 1:** Additional sample characteristics

*Professional status*	
Student	45%
Working	46%
Retired	5%
Unemployed	1%
Other	3%
*Highest level of education* (*for N* = *256 non*-*students*)	
Apprenticeship	22%
University degree	15%
Other higher education/professional degree	56%
Other	7%
*Current work life characteristics due to Covid*-*19* (*multiple choice allowed* (for *N *=* 256 non*-*students*))	
Professional situation has not changed	25%
I now work in home office	46%
I had to temporarily give up work	15%
I permanently lost my job	3%
I have to work more than usual	42%
*Other characteristics*	
I am in a permanent romantic relationship	42%
I have children (*if yes*: at least one child currently lives in my household)	21% (44%)
People living in household	*M* = 3.0 (*SD* = 1.4)

*N* = 466 unless indicated otherwise

### Instruments

#### Loneliness

Loneliness was assessed with the German short version of the University of California Los Angeles (UCLA) Loneliness Scale (Luhmann et al. 2016). This nine-item version consists of three items on intimate loneliness (e.g. “How often do you feel that you lack companionship?”), relational loneliness (e.g. “How often do you feel that there are people you can talk to?”), and collective loneliness (e.g. “How often do you feel that you have a lot in common with the people around you?”), respectively. For each item, participants rated on a four-point Likert scale (never, rarely, sometimes, always) their experience of loneliness in the last 7 days, yielding one total mean score of loneliness across all items.

#### Well-Being

A German version of the WHO-5 index (Brähler et al. 2007) was used to measure the affective component of well-being. Participants rated how often they had felt in “good spirits”, “active”, “relaxed” etc. for the last 7 days on five items using a 7-point Likert scale from “never” to “always”, yielding one mean well-being score.

#### State Anxiety and State Depression

For the assessment of state anxiety and state depression the state questionnaire from the State-Trait Anxiety-Depression Inventory (STADI; Laux et al. 2013) was used. This scale consists of four subscales with five items each. Two subscales refer to emotionality (e.g. “I am nervous”) and worry (e.g. “I am unsure if everything will go well”) as facets of anxiety and two subscales refer to euthymia (positive affect and experience of happiness, e.g. “I am in a good mood”) and dysthymia (negative affect and depressive mood, e.g. “I am unhappy”). Participants indicated their agreement with each statement for the last 7 days on a 4-point Likert scale from “not at all” (1) to “very much” (4). An anxiety score was computed as the mean value across the emotionality and worry items. A depression score was computed as the mean across the recorded dysthymia and the inverted euthymia items.

#### Extraversion and Neuroticism

The German translation of the two extraversion and neuroticism scales from the International Personality Item Pool (IPIP) was used (Ostendorf 2003). Extraversion (e.g. “I am the life of the party”) and neuroticism (e.g. “I get stressed out easily”) consist of 10 items each to be rated on a scale from 1 (very inaccurate) to 5 (very accurate). Mean scores for extraversion and neuroticism items were computed across the 10 items, respectively.

#### Cognitive Emotion Regulation

The German short version of the Cognitive Emotion Regulation Questionnaire (CERQ-short; Loch et al. 2011) consists of 18 items assigned to the five adaptive regulation subscales acceptance (e.g. “I think that I have to accept that this has happened”), positive refocusing (e.g. I think of pleasant things that have nothing to do with it”), refocus on planning (e.g. “I think about a plan of what I can do best”), positive reappraisal (e.g. “I think I can learn something from the situation”), putting into perspective (e.g. “I tell myself that there are worse things in life”) and to the four maladaptive regulation subscales catastrophizing (e.g. “I keep thinking about how terrible it is what I have experienced”), other-blame (e.g. “I feel that others are responsible for what has happened”), self-blame (e.g. “I feel that I am the one who is responsible for what has happened”), and rumination (e.g. “I am preoccupied with what I think and feel about what I have experienced”). Participants were asked to indicate how often they think in ways reflecting each strategy after the experience of threatening or stressful life events on a 5-point Likert scale from “(almost) never” to “(almost) always”. An adaptive and a maladaptive regulation score were computed as the means of all items belonging to the respective set of strategies.

#### Suppression of Emotions

From the German version of the Emotion Regulation Questionnaire (Abler and Kessler 2009), the suppression subscale (e.g. “When I am feeling negative emotions, I make sure not to express them”) was used to complement the cognitive CERQ strategies with a behavioral regulation strategy, namely suppressing the expression of one’s own emotions to others. Participants rated the four items on a 7-point Likert scale from “strongly disagree” to “strongly agree” and the mean of these items yielded the total suppression score.

#### Other Variables

The present study was part of a larger project that included measures of other personality traits (including conscientiousness and aggression) and of Covid-19 specific behaviors (e.g., adhering to social distance rules, media consumption), which are not analyzed here.

### Data Analysis

Prior to hypothesis testing, the WHO-5 well-being scale and STADI anxiety and depression subscales were combined into a well-being composite score representing affective aspects of well-being (see Results section). To examine whether personality, cognitive emotion regulation strategies, and time were related to loneliness and well-being, two hierarchical regression analyses were performed with loneliness and the well-being composite as dependent variables. In a first step, the control variables age, gender, and relationship status were included in the model as independent variables. In a second step, number of days since lockdown (based on the date a participant completed the study), extraversion, neuroticism, adaptive and maladaptive cognitive strategies as well as suppression of emotions were included as independent variables. A higher number of days since lockdown indicated more time spent under public life restrictions at the time of study participation.

For exploratory purposes, two-way interaction terms between all independent variables were included into the two regression analyses in a third step (e.g., Aiken and West 1991). For the sake of parsimony, models were further adjusted through stepwise removal of statistically non-significant two-way interactions. This process continued until only significant two-way interactions remained in the models. Fit indices (*χ*^*2*^, Akaike Information Criterion [AIC], and Bayesian Information Criterion [BIC]) were then compared between the models with all interactions and the model with only significant interactions. As the models with all interactions did not show better fit than the model with only significant interactions (see results section), the more parsimonious models are reported. All variables were *z* standardized before they were entered into the regression analyses.

In order to test whether results were biased by the large number of students in the sample, we additionally re-ran all models with a binary control variable (students vs. non-students). Variance explained as well as all regression coefficients remained virtually unchanged, suggesting that results were not driven by one of the subsamples. Below, the models without this variable are reported.

A power analysis performed with G*Power revealed a necessary *N* of 347 to detect small to medium effect size (f^2^ = .10) with a power of .95 and an alpha level of .05 in a multiple regression analyses with 24 variables (all assessed predictors including all control variables, main effects and interactions). Therefore, the study was well-powered. The data and the analysis script can be found here: https://osf.io/nekfu/?view_only=9e5e80c3409c401cbef71c8138178e58.

## Results

Means, standard deviations and internal consistency (Cronbach’s alpha) of all measures are presented in Table 2. The anxiety subscale and depression STADI subscales correlated at *r* =  .59, *p* < .001. The WHO-5 scale correlated significantly with the anxiety subscale, *r* = − .53, *p* < .001, and with the depression subscale, *r* = − .65, *p* < .001 (see Table 3). We therefore tested whether these three measures could be combined into one score by conducting a principal component analysis. The Bartlett test, χ^2^(3) = 476.26, *p* < .001, as well as the Kaiser–Meyer–Olkin (KMO) measure of sampling adequacy, KMO = 0.7, showed that these three variables were suitable for factor analysis. A principal component analysis with Varimax rotation showed that a one-factor solution explained 59.4% of the variance. The factor loadings of the three scales were therefore combined into a total well-being score with higher values indicating higher well-being (anxiety and depression scores were reversed).

**Table 2:** Means (M), standard deviations (SD), and Cronbach’s alpha for all questionnaires

	M	SD	α
IPIP extraversion	3.25	0.73	.88
IPIP neuroticism	2.8	0.78	.89
CERQ adaptive	3.5	0.56	.74
CERQ maladaptive	2.53	0.56	.69
ERQ suppression	3.53	1.19	.75
UCLA loneliness	1.92	0.41	.78
STADI anxiety	1.88	0.51	.87
STADI depression	1.93	0.53	.91
WHO-5 well-being	3.67	1.00	.85

*IPIP* International Personality Item Pool, *CERQ* Cognitive Emotion Regulation Questionnaire, *ERQ* Emotion Regulation Questionnaire, *UCLA* University of California Los Angeles, *STADI* State-Trait Anxiety-Depression Inventory, *WHO* World Health Organization. *N* = 466

**Table 3:** Zero-order correlations for all variables included in the multiple linear regressions

	1. Time	2.	3.	4.	5.	6.	7.	8.	9.	10.	11.
2. Age	.08										
3. Gender	− .01	− .14**									
4. Relationship status	− .01	− .32***	.04								
5. Students versus non-students	− .04	.53***	− .08	− .22***							
6. IPIP extraversion	.04	.02	.00	.06	.07						
7. IPIP neuroticism	− .08	− .32***	.18***	.00	− .20***	− .26***					
8. CERQ adaptive	.00	.04	.05	.02	.00	.13**	− .26***				
9. CERQ maladaptive	− .02	− .36***	.13**	.11*	− .24***	− .11*	.53***	− .07			
10. ERQ suppression	.03	− .01	− .13**	− .11*	.04	− .29***	.02	− .03	.02		
11. Loneliness	− .05	− .17***	− .02	− .10*	− .08	− .17***	.29***	− .19***	.21***	.25***	
12. Well-being	.13**	.28***	− .17***	.05	.16***	.16***	− .57***	.22***	− .36***	− .06	− .49***

*N* = 466. For gender 0 = male, 1 = female, for relationship status 0 = single, divorced or widowed, 1 = in a romantic relationship, for students versus non-students 0 = student, 1 = non-student. *IPIP* International Personality Item Pool, *CERQ* Cognitive Emotion Regulation Questionnaire, *ERQ* Emotion Regulation Questionnaire

*p < .05, **p < .01, ***p < .001

Zero-order correlations for all variables are provided in Table 3. As can be seen in Table 3, extraversion was positively associated with well-being and negatively with loneliness; however, the effect size was small. Neuroticism showed a moderate positive association with loneliness and a strong negative association with well-being. Extraversion and neuroticism were not related to time (days since lockdown at the time of study participation), but they were negatively related to each other to a moderate degree. Adaptive emotion regulation strategies were positively related to well-being and negatively related to loneliness, while maladaptive strategies were negatively related to well-being and positively to loneliness. Correlations were small to moderate. Although suppression of emotions was not associated with well-being, it was positively related to loneliness. The three regulation scores (adaptive, maladaptive, suppression) were uncorrelated with each other. Time was positively associated with well-being but not with loneliness. Regarding emotion regulation strategies and personality, neuroticism was positively related to maladaptive strategies and negatively to adaptive strategies but not to the suppression of emotions. Extraversion, in turn, was negatively related to maladaptive strategies and positively to adaptive strategies. However, extraversion showed the strongest association with suppression of emotions.

**Fig. 1:**
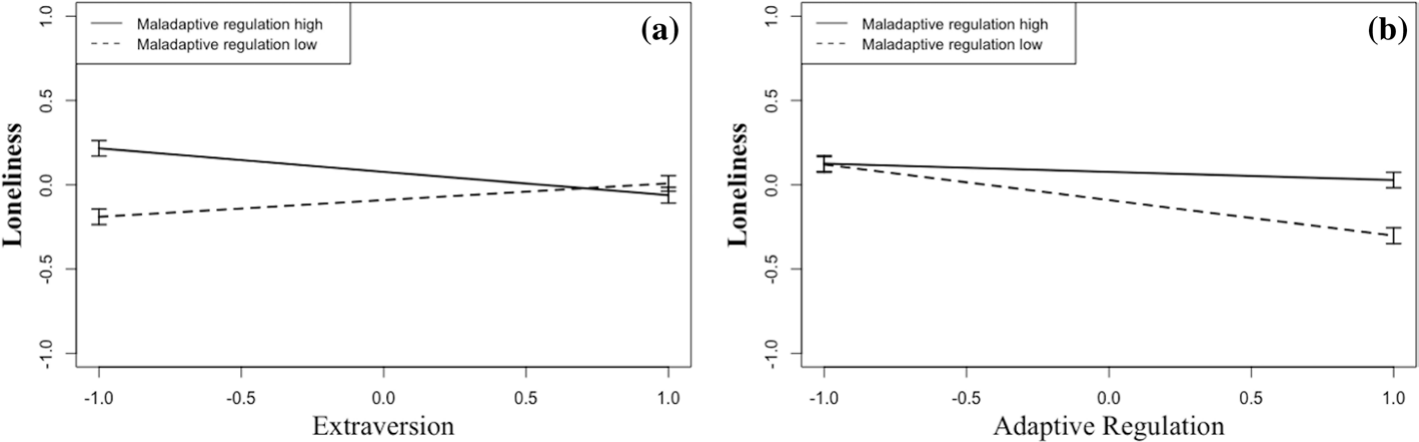
Left (**a**) loneliness under public life restrictions as a function of extraversion and maladaptive emotion regulation strategies. Right (**b**) loneliness under public life restrictions as a function of adaptive and maladaptive emotion regulation strategies

The results of the hierarchical multiple regression predicting loneliness are presented in Table 4. In the first step, control variables age, gender and relationship status were entered and revealed significant main effects for age and relationship status (*p *< .001), which explained 5% of variance in loneliness. Older participants and those in a romantic relationship reported feeling less lonely. In the second step, main effects for time (days since lockdown at the time of study participation), extraversion, neuroticism, and the three emotion regulation scores were included. Time, extraversion, and maladaptive regulation did not explain unique portions of variance in loneliness. Neuroticism and suppression of emotions, however, were positively and adaptive regulation strategies were negatively associated with loneliness. The second step explained an additional 12% of the variance in loneliness. Finally, all possible two-way interactions between the independent variables were entered into the regression. The model was adjusted by stepwise removing all statistically non-significant interactions. Two significant interaction terms emerged and appear in the final model. The model without non-significant interactions fitted the data not significantly worse than the model with all interaction terms, *Δ*χ2 (13) = 5.74, *p* = .905, but described the data more parsimoniously as indicated by AIC and BIC. AIC (1241.795) and BIC (1295.670) were smaller for the model without non-significant interactions than for the model with all interaction terms (AIC = 1260.522, BIC = 1368.271). As can be seen in Table 4, the interaction effect between extraversion and maladaptive regulation strategies reached statistical significance. As illustrated in Fig. 1a, the use of maladaptive strategies was associated with loneliness only in introverts but not in extraverts. In introverts (participants with low extraversion), low use of maladaptive strategies was associated with lower loneliness and high use of maladaptive strategies was associated with higher loneliness.

**Table 4:** Hierarchical regression with loneliness as dependent variable

	Dependent variable: loneliness				
	B(CI)	SE B	β	t-value	*p* value
*Step 1*					
(Intercept)	.00 (− .09; .09)	.05		.00	
Age	− .23 (− .33; − .14)	.05	− .23	− 4.83	.000***
Gender	− .04 (− .13; .05)	.05	− .04	− .96	.336
Relationship status	− .17 (− .27; − .08)	.05	− .17	− 3.66	.000***
	*F*(3) = 9.42, Adjusted *R*^2^ = .05, *p* < .001				
*Step 2*					
(Intercept)	.00 (− .08; .08)	.04		.00	
Age	− .12 (− .22; − .03)	.05	− .12	− 2.50	.012*
Gender	− .04 (− .12; .05)	.04	− .04	− .86	.393
Relationship status	− .12 (− .21; − .03)	.05	− .12	− 2.65	.008**
Time	− .03 (− .12; .05)	.04	− .03	− .79	.428
Extraversion	− .03 (− .12; .06)	.05	− .03	− .63	.527
Neuroticism	.17 (.07; .28)	.06	.17	3.17	.001**
Adaptive strategies	− .12 (− .21; − .03)	.04	− .12	− 2.74	.006**
Maladaptive strategies	.08 (− .03; .18)	.05	.08	1.46	.143
Suppression of emotions	.21 (.13; .30)	.04	.21	4.75	.000***
	*F*(9) = 11.41, Adjusted *R*^2^ = .17, *ΔR*^*2*^ = .12, *p* < .001				
*Step 3*					
(Intercept)	− .01 (− .09; .08)	.04		− .17	
Age	− .13 (− .23; − .04)	.05	− .13	− 2.7	.007**
Gender	− .04 (− .13; .04)	.04	− .04	− .95	.341
Relationship status	− .12 (− .21: − .03)	.04	− .12	− 2.70	.007**
Time	− .03 (− .11; .06)	.04	− .03	− .62	.535
Extraversion	− .02 (− .11; .07)	.05	− .02	− .43	.663
Neuroticism	.18 (.07; .28)	.05	.18	3.23	.001**
Adaptive strategies	− .13 (− .22; − .04)	.04	− .13	− 2.95	.003**
Maladaptive strategies	.08 (− .02; .19)	.05	.08	1.61	.107
Suppression of emotions	.21 (.12; .30)	.04	.21	4.77	.000***
Extraversion * Maladaptive strategies	− .12 (− .20; − .04)	.04	− .12	− 2.84	.004**
Adaptive strategies * Maladaptive strategies	.08 (.00; .16)	.04	.08	1.983	.048*
	*F*(11) = 10.50, Adjusted *R*^2^ = .18, *ΔR*^*2*^ = .01, *p* < .001				

*N* = 466. B = unstandardized regression weight, CI = 95% Confidence Interval of unstandardized regression weight (B), SE B = Standard Error of unstandardized regression weight (B), β = standardized regression weight. For gender, 0 = male, 1 = female; for relationship status, 0 = single, divorced, or widowed, 1 = in a romantic relationship

*p < .05, **p < .01, ***p < .001

The second significant interaction effect was found for adaptive and maladaptive regulation strategies, indicating the lowest loneliness levels were reported by participants that scored high on adaptive regulation and low on maladaptive regulation (Fig. 1b). Altogether, 18% of the variance of loneliness were explained by the independent variables and the interaction terms. The moderations alone explained 1% more variance than the second step without interactions.

**Fig. 2:**
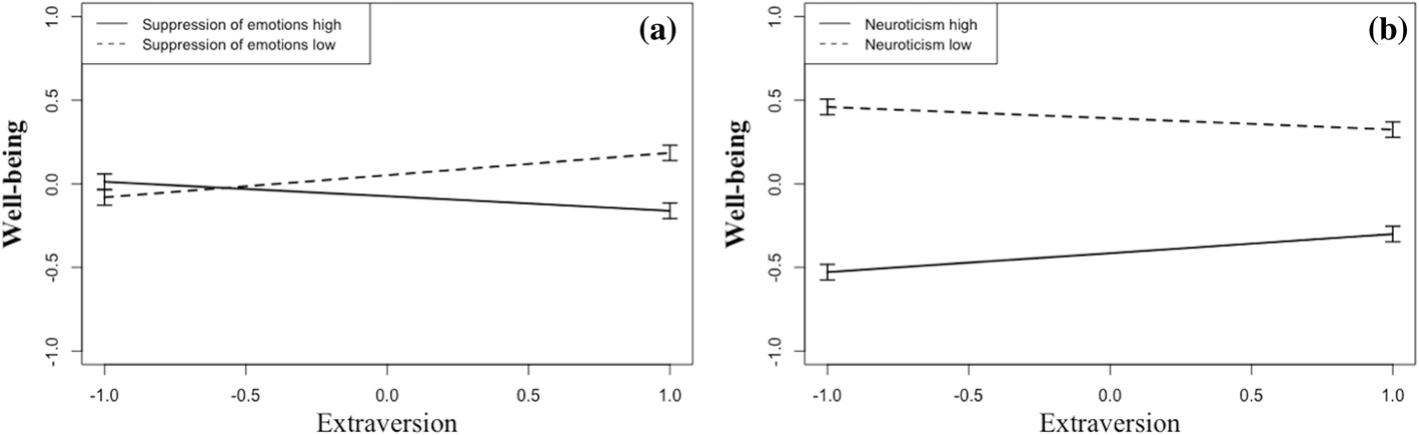
Left **a** Well-being under public life restrictions as a function of extraversion and the level of suppression of emotions. Right **b** Well-being under public life restrictions as a function of extraversion and neuroticism

**Fig. 3:**
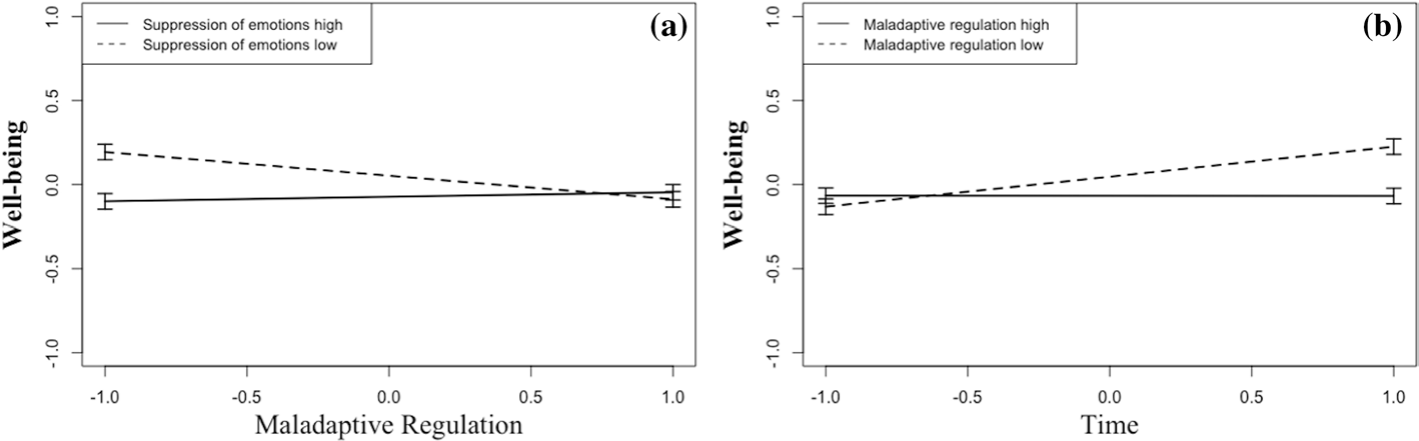
Left **a** Well-being under public life restrictions as a function of maladaptive regulation and suppression of emotions. Right **b** Well-being under public life restrictions as a function of time and maladaptive emotion regulation

The results of the second hierarchical regression analysis predicting the well-being composite are presented in Table 5. In the first step, age, gender, and relationship status explained 11% of the variance, with older participants, males, and those in a romantic relationship reporting higher well-being. In the second step, main effects for all independent variables were entered. Extraversion, maladaptive regulation and suppression of emotions were not uniquely associated with well-being. However, neuroticism was strongly negatively linked, and adaptive emotion regulation was positively linked, to well-being. Furthermore, participants that were tested later in time (after more days in lockdown) reported higher well-being. The second step explained an additional 24% of the variance in well-being. In the third step, all possible two-way interactions were entered into the regression. The model was further adjusted by stepwise removing all statistically non-significant interactions. Four significant interactions emerged and appear in the final model. The model without non-significant interactions fitted the data not significantly worse than the model with all interaction terms, *Δ*χ2 (10) = 1.62, *p* = .977, but described the data more parsimoniously as indicated by AIC and BIC. AIC (1020.494) and BIC (1086.800) were smaller for the model without non-significant interactions than for the model with all interaction terms (AIC = 1037.162, BIC = 1144.911). The significant interaction effects are illustrated in Figs. 2 and 3. As displayed in Fig. 2a, extraverts with low emotional suppression showed higher well-being than extraverts with high emotional suppression. However, suppression of emotions hardly affected the well-being of introverts. The second interaction showed that neuroticism affected well-being more strongly in introverts than in extraverts (Fig. 2b). The third interaction revealed that low emotion suppression was only beneficial to the well-being of individuals who reported low levels of maladaptive regulation (Fig. 3a). The last interaction indicated that individuals who participated at a later point in time reported higher well-being, but only if they scored low on maladaptive regulation (see Fig. 3b). Overall, the regression model explained 39% of the variance in well-being, with the interactions adding 4% explained variance to the main effects.

**Table 5:** Hierarchical regression with well-being as dependent variable

	Dependent Variable: Wellbeing				
	B(CI)	SE B	β	t-value	*p* value
*Step 1*					
(Intercept)	.00 (− .08; .08)	.04		.00	
Age	.27 (.19; .36)	.04	.30	6.42	.000***
Gender	− .11 (− .19; − .03)	.04	− .12	− 2.82	.005**
Relationship status	.15 (.07; .24)	.04	.17	3.68	.000***
	*F*(3) = 19.20, Adjusted *R*^*2*^ = .11, *p* < .001				
*Step 2*					
(Intercept)	.00 (−.07; .07)	.05		.00	
Age	.10 (.02; .18)	.04	.11	2.59	.009**
Gender	− .07 (− .14; .00)	.04	− .07	− 1.94	.053
Relationship status	.09 (.02; .17)	.03	.10	2.60	.009**
Time	.08 (.02; .15)	.03	.09	2.42	.015*
Extraversion	.01 (− .06; .08)	.04	.01	.23	.817
Neuroticism	− .41 (− .50; − .32)	.04	− .45	− 9.25	.000***
Adaptive strategies	.10 (.03; .17)	.04	.11	2.93	.003**
Maladaptive strategies	− .05 (− .13; .03)	.04	− .05	− 1.19	.235
Suppression of emotions	− .05 (− .12; .02)	.04	− .06	− 1.45	.147
	*F*(9) = 29.36, Adjusted *R*^*2*^ = .35, *ΔR*^*2*^ = .24, *p* < .001				
*Step 3*					
(Intercept)	− .01 (− .08; .06)	.04		− .30	
Age	.09 (.02; .17)	.04	.10	2.44	.037*
Gender	− .07 (− .14; .00)	.03	− .08	− 2.01	.045*
Relationship status	.10 (.03; .17)	.04	.11	2.75	.006**
Time	.09 (.02; .15)	.03	.10	2.68	.007**
Extraversion	.02 (− .05; .09)	.04	.03	.64	.538
Neuroticism	− .40 (− .49; − .32)	.04	− .44	− 9.35	.000***
Adaptive strategies	.08 (.01; .15)	.03	.09	2.37	.018*
Maladaptive strategies	− .06 (− .14; .02)	.04	− .06	− 1.41	.162
Suppression of emotions	− .06 (− .13; .01)	.04	− .07	− 1.80	.071
Time* Maladaptive strategies	− .09 (− .16; − .02)	.03	− .10	− 2.64	.008**
Extraversion * Neuroticism	.09 (.02; .16)	.03	.10	2.68	.007**
Extraversion * Suppression of emotions	− .11 (− .17; − .05)	.03	− .12	− 3.38	.000***
Maladaptive strategies * Suppression of emotions	.08 (.01; .15)	.04	.09	2.37	.018*
	*F*(13) = 24.14, Adjusted *R*^*2*^ = .39, *ΔR*^2^ = .04, *p* < .001				

*N* = 466. B = unstandardized regression weight, CI = 95% confidence interval of unstandardized regression weight (B), SE B = standard error of unstandardized regression weight (B), β = standardized regression weight. For gender, 0 = male, 1 = female; for relationship status, 0 = single, divorced, or widowed, 1 = in a romantic relationship

*p < .05, **p < .01, ***p < .001

## Discussion

Past research established neuroticism, extraversion, and emotion regulation styles as important correlates and predictors of loneliness and well-being. However, little is known about how these traits—particularly extraversion—are related to psychological well-being during a global health crisis that is marked by drastic public life restrictions, stress, and worries for many people around the world. Furthermore, previous studies rarely examined possible interaction effects of personality and emotion regulation. The present study therefore aimed to investigate how neuroticism, extraversion, and emotion regulation styles—uniquely and in combination—are associated with loneliness and well-being over the course of 6 weeks of Covid-19-related social distancing restrictions in Switzerland. Loneliness and well-being measures both referred to the last 7 days and well-being was defined in terms of low anxiety, low depressive symptoms, and high levels of enjoyment and energy.

Regarding the direct role of personality traits, neuroticism was most strongly related to loneliness and reduced well-being in line with our expectations. The effect sizes in the present study were comparable to the ones found in the meta-analysis of Buecker et al. (2020) for loneliness (medium effects) and in the meta-analyses of Albuquerque et al. (2012) and Steel et al. (2008) for well-being (high effects). This suggests that emotional stability is equally associated with psychological outcomes under “normal” circumstances and in an exceptional global crisis affecting most humans. These findings are consistent with the notion that individuals high in neuroticism react to challenging situations with more fear and distress, are more sensitive to cues of rejection, and show dysfunctional interpersonal behavior in close relationships (Denissen and Penke 2008; Vater and Schröder-Abé 2015)—factors that likely also came into play when facing the consequences of Covid-19-related public life restrictions as was shown by other recent studies (Aschwanden et al. 2020; Fernández et al. 2020; Kroencke et al. 2020; Liu et al. 2021).

For extraversion, however, results showed a quite different picture in comparison to studies conducted outside the context of crises. Notably, the zero-order correlations with loneliness and well-being went into the expected directions but were much smaller in magnitude than those obtained in other studies (e.g., Buecker et al. 2020; Steel et al. 2008). In the multiple regression analyses, no significant main effects of extraversion were found. This might be due to the significant correlations of extraversion with neuroticism and adaptive regulation, which were in turn more highly correlated with loneliness and well-being than extraversion (see Table 2). Thus, the zero-order variance explained by extraversion might have been mainly driven by its associations with neuroticism and adaptive regulation.

These results are in contrast to the high negative meta-analytic correlation between extraversion and loneliness even after controlling for other personality traits (Buecker et al. 2020) and the moderate to high positive meta-analytic correlation between extraversion and well-being (Steel et al. 2008; DeNeve and Cooper 1998). However, they are compatible with a recent Covid-19 study in which higher extraversion was related to greater health- and economy-related concerns during the pandemic (Aschwanden et al. 2020). One possible interpretation—assuming a causal relation between personality and wellbeing—is that extraversion loses some of its protective value for mental health during phases of drastic and imposed public and social life restrictions. This is in line with previous research suggesting that extraverts have higher well-being because they seek the company of others, engage in social activities, and interact with others for support (Buecker et al. 2020)—activities which became temporarily less available both in private life (meeting friends and family) and in the work context (talking to colleagues). Consequently, extraverts may have felt more burdened and lonelier than usual, decreasing the overall association between extraversion and psychological well-being. However, it is necessary to stress that the present study was cross-sectional and did not investigate causal relationships among personality, loneliness, and well-being.

With respect to the direct role of emotion regulation, adaptive strategies were significantly associated with both higher well-being and lower loneliness and suppression of emotions was significantly associated with higher loneliness. These findings are in line with our predictions and with previous research showing that individuals who suppress their emotions tend to communicate less frequently with others, have weaker social ties and, consequently, less social support (Butler et al. 2003; Gross and John 2003; Srivastava et al. 2008). Adaptive strategies such as acceptance, focusing on planning, putting into perspective or reappraisal, on the other hand, might have helped to see the public life restrictions in a more positive light, to find ways to use this time in a more productive way, and to actively seek social support through different ways (e.g., through social media). These strategies may have helped maintaining positive emotions which may have positively affected well-being (Troy and Mauss 2011). Again, although the present findings are consistent with such causal interpretations, the present study only assessed cross-sectional associations. Maladaptive cognitive strategies such as rumination and catastrophizing were not related to loneliness and well-being in the multiple regressions, which may be due to the high overlap with neuroticism.

Regarding interactions between personality and emotion regulation, we could confirm the expectation that extraversion would show more interaction effects than neuroticism because extraversion and regulation styles show only small to moderate associations (see Table 3). Specifically, we found that in introverts, maladaptive regulation was related to loneliness (Fig. 1a), and in extraverts, suppression was associated with well-being (Fig. 2a). Introverts, but not extraverts, profited from low maladaptive regulation in that they reported lower loneliness. Conversely, extraverts but not introverts profited from low expressive suppression in that they reported higher well-being.

Compared to extraverts, introverts are more focused on their inner world (Hills and Argyle 2001; Jung 1928). Their subjective feelings of loneliness and well-being may therefore primarily depend on internal emotional processes, whereas for extraverts the quality and quantity of social interactions are more important (Hills and Argyle 2001). This might explain why internal cognitive strategies that are focused on changing the meaning of an emotional event (e.g., rumination, self-blame, or catastrophizing) are more important in introverts, whereas the behavioral response-focused strategy of suppressing one’s feelings to others is more important in extraverts (Gross and John 2003). In addition, suppression relates to less social support, which is associated with more distress in extraverts than introverts (Butler et al. 2003; Gross and John 2003; Srivastava et al. 2009; Duckitt 1984). The suppression of emotions might therefore have a particularly negative effect on extraverts’ well-being. The well-being of introverts, on the other hand, might not depend on how strongly they suppress their emotions because they rely less on social support.

Taken together, these exploratory findings provide support for the assumption that complex interactions between extraversion and various emotion regulation strategies do exist and should be further examined in future studies. The present findings can help generating more detailed theory-driven predictions for future research, although it remains unclear at this point whether extraversion and emotion regulation will interact in the same way outside of the exceptional circumstances of the Covid-19 pandemic. It should also be noted that the interaction effects explained only small portions of the variance.

With respect to interactions among emotion regulation styles, we found that (1) the combination of low maladaptive regulation and high adaptive regulation was associated with the lowest loneliness scores (Fig. 1b), and (2) the combination of low maladaptive regulation and low suppression was associated with the highest well-being scores (Fig. 3a). These results are consistent with Dixon-Gordon et al. (2015) who found that a balanced regulation profile with a higher use of adaptive as compared to maladaptive strategies (including cognitive strategies as well as expressive suppression) went along with lower levels of psychopathology than other profiles (e.g., the “high regulator profile” with habitual use of both adaptive and maladaptive strategies). These authors argued that the simultaneous frequent use of adaptive and maladaptive strategies might reflect an inflexible, indiscriminate, regulation style. The present findings underscore the importance of assessing a person’s regulation style using a large set of cognitive and behavioral strategies and by examining profiles or “repertoires” rather than single strategies (Dixon-Gordon et al. 2015).

The final hypothesis we examined postulated that participants who were tested at a later time point during the drastic imposed public life restrictions in Switzerland would show lower well-being and more loneliness because the negative social consequences would accumulate as found in quarantine studies (Brooks et al. 2020; Hawryluck et al. 2004). However, our data showed the opposite effect, at least for well-being: For individuals with low maladaptive regulation, well-being increased the later they participated in the study (Fig. 3b). For individuals with high maladaptive regulation, the date of participation was unrelated to well-being.

The discrepancy of this finding is likely due to the fact that quarantine is much more restrictive than the social distancing measures implemented in Switzerland. In Switzerland, unlike in some other countries or regions, people were always allowed to leave their homes. Furthermore, the Covid-19 pandemic in Switzerland turned out to be less severe than expected during the first months, and the health system did not collapse (Federal Office of Public Health 2020). As a result, the initial fear and uncertainty at the beginning of the pandemic might have been replaced by hope and confidence, at least among people who are not prone to rumination or catastrophizing. Because countries differ widely in the implemented distancing measures, the progression of the pandemic, the national economic and social consequences, and the burden on the public healthcare system, more research is needed to examine the mid- and long-term effects of public life restrictions on people’s well-being and mental health. Preferably, such studies should use longitudinal designs (e.g., Kwong et al. 2020) and should be conducted across countries (e.g., Ammar et al. 2020).

There are several limitations to the present study. First, as mentioned above, the results were obtained in Switzerland and should be replicated in other countries. Second, the study was cross-sectional and the development of well-being and loneliness over time was assessed by using the date of study participating as a predictor. Although age, gender, and relationship status were controlled for, it may be that other factors drove the respective findings (e.g., lower motivation of people with low well-being to participate at later dates). Third, although the present study covered a wide age range, the sample was not representative of the general population. A large portion of participants (about 45%) were psychology students and about 80% were female. Fourth, we did not investigate the mechanisms underlying the present findings and especially the explanations for the exploratory results remain speculative. It would therefore be important to substantiate the current results in future studies by measuring concrete Covid-19-related thoughts, feelings, and behaviors, especially regarding social support.

Despite these limitations, the present study provided important insights into the correlates of loneliness and well-being during a global health crisis and revealed potentially fruitful avenues for future research into personality, emotion regulation, and mental health more generally. Although not of primary importance for the present research questions, demographic variables emerged as significant predictors. Specifically, older participants and participants in a romantic relationship reported higher well-being and lower loneliness, and men reported higher well-being than women, which is generally in line with previous research (e.g., Diener et al. 2000). With respect to the personality correlates of loneliness and well-being, neuroticism unsurprisingly emerged as the most important one. Furthermore, adaptive emotion regulation and emotional suppression uniquely related to these outcomes in the expected directions. These findings raise the question of whether people with low levels of adaptive regulation might benefit from (web-based) training programs promoting a healthy emotion regulation style, specifically during globally challenging crises (e.g., Glück and Maercker 2011).

More surprising, however, was the finding that extraversion showed only small associations with these outcomes and that these associations were moderated by different emotion regulation styles for introverts and extraverts. While under “normal” circumstances, extraversion is considered a unique and strong correlate and predictor of lower loneliness and better well-being, the temporary imposed restriction of possibilities to maintain (physical) social contact with others may have weakened the protective character of extraversion.

Finally, with respect to future directions for personality research, the present study suggested that it is worthwhile to examine interaction effects among personality traits and different emotion regulation strategies, as these strategies may moderate the relationship with psychological well-being. Furthermore, our study implied that personality may not uniformly relate to well-being, but that the associations may change depending on specific life events or environmental circumstances.
